# A Giant Primary Mediastinal Teratocarcinoma in a Male Adult

**DOI:** 10.1155/2019/7123241

**Published:** 2019-06-10

**Authors:** Muneera Al-Khalifa, Sara Alsaad, Habib Al-Tareef, Zaid Arekat, Abdulla Darwish

**Affiliations:** ^1^RCSI-MUB, Busaiteen, Bahrain; ^2^Department of Pathology, Bahrain Defense Force Hospital, Riffa, Bahrain; ^3^Mohammed Bin Khalifa Cardiac Center, Bahrain Defense Force Hospital, Riffa, Bahrain

## Abstract

Germ cell tumors (GCTs) arise along the midline, in which 50-70% of extragonadal GCTs occur in the mediastinum. Malignant GCTs are more common in males, while benign GCTs occur equally in both males and females. This report presents a case of a giant primary mediastinal nonseminomatous GCT resected from a 35-year-old male who presented with dyspnoea and tightness in the chest. Thorough investigations including a chest MRI were done. It showed a 21 × 19 × 15 cm tumor. Thus, surgical resection of the tumor through a midline sternotomy was done. Histopathological analysis diagnosed the tumor as a primary mediastinal teratocarcinoma with a sarcomatous component. Eighteen-month follow-up showed no tumor recurrence. Mediastinal teratocarcinoma is a rare and life-threatening germ cell tumor. Studies recommend the use of chemotherapy prior to resection as an important step in its management. Close and regular follow-up postsurgical resection is advised.

## 1. Introduction

Germ cell tumors (GCTs) arise along the midline across from the pineal gland to the presacral area [1, 2]. They form due to the incomplete migration of primitive germ cells during the early stage of embryonic development [1]. Most GCTs arise in a gonadal tissue; however, 50-70% of extragonadal GCTs occur in the mediastinum [1, 3]. GCTs are broadly classified as either the teratomatous or nonteratomatous type [1].

Benign GCTs have no gender predilection, and they account for 70-80% of mediastinal GCTs [1, 3]. Malignant GCTs, on the other hand, occur more frequently in males [1, 4, 5].

GCTs should be differentiated from other anterior mediastinal tumors, which could be benign or malignant neoplasms [2]. These tumors include thymic tumors and cysts, thyroid lesions, parathyroid adenomas, malignant lymphomas, paragangliomas, lymphangiomas, hemangiomas, or lipomas [2]. The diagnoses of these tumors are usually straightforward, but in difficult cases, immunohistochemistry studies play an important diagnostic role.

Tumors in the mediastinum can be life threatening because they grow in a confined space between the lungs. People with mediastinal tumors can be asymptomatic but are most likely to present with symptoms of mediastinal obstruction, such as dyspnoea, dysphagia, and chest pain [1, 3, 4, 6].

Neoadjuvant chemotherapy preceding surgical resection is recommended in patients with NSGCTs. Studies recommend using a cisplatin-based chemotherapy regimen for NSGCT as patients' demonstrated better outcome [3].

## 2. Case Presentation

**Figure 1:**
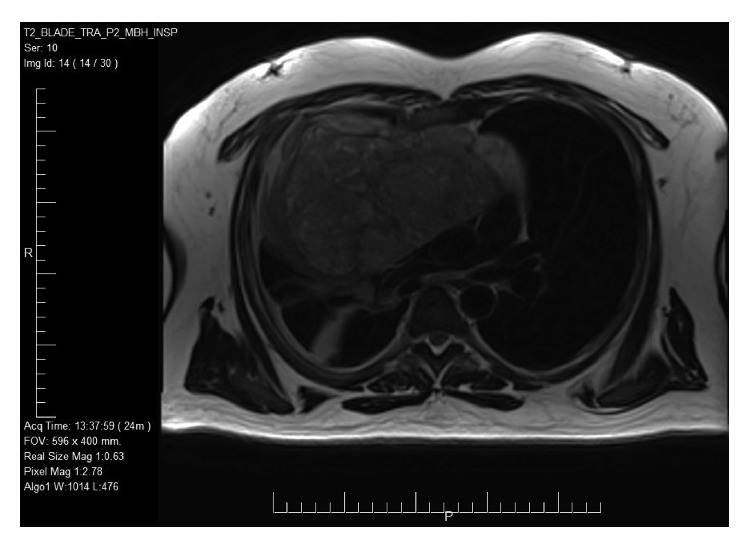
A large mediastinal mass is noted on MRI before surgery.

A 35-year-old male presented to a secondary healthcare center with shortness of breath and chest tightness. A chest X-ray was done and showed left pleural effusion. The pleural fluid was drained and sent to the Pathology Department for further analysis. It showed malignant cells. A CT scan of the chest was then requested and revealed a heterogeneous anterior mediastinal mass. In addition, a chest MRI was performed and it showed a well-defined, lobulated, and heterogeneous anterior mediastinal mass measuring 15.9 × 15 × 14.5 cm occupying the right hemithorax (Figure 1). This mass was compressing the adjacent structures and causing compressive atelectasis of the anterior segment of the right upper lobe. However, the mediastinal mass did not show any signs of direct invasion. A scrotal ultrasound was performed, and it revealed bilateral varicocele; however, there was no evidence of testicular mass.

A Tru-Cut biopsy was performed, and histopathological examination showed features of an undifferentiated malignant tumor. Immunohistochemistry revealed the following profile: the tumor cells were strongly positive for AFP, vimentin, and OCT3/4 and focally positive for CD99, CK7, and p63. The tumor cells were negative for CD30, PLAP, TTF1, HCG, synaptophysin, chromogranin, WT1, and calretinin. The Ki-67 proliferation index was almost 80%. Overall, the appearances were consistent with a nonseminomatous germ cell tumor (NSGCT) in keeping with a yolk sac tumor.

**Figure 2:**
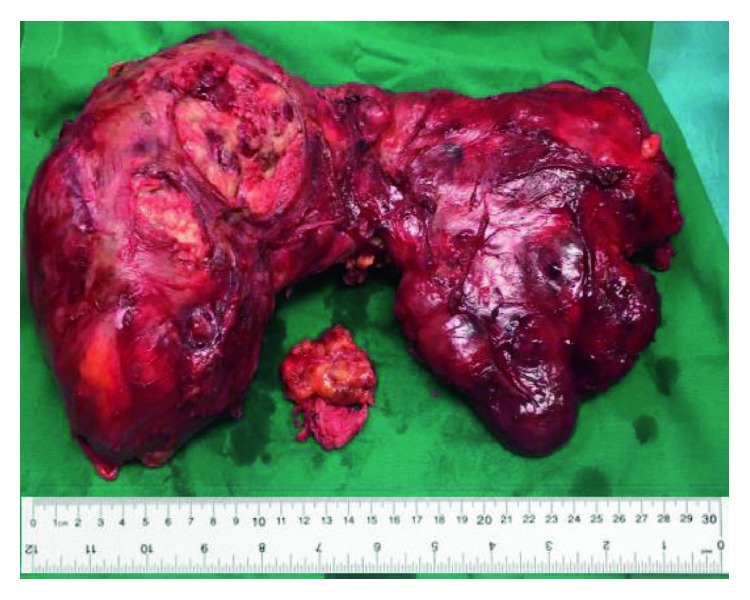
The resected tumor next to a 30 cm ruler.

The patient was referred to a tertiary healthcare center. Another chest MRI was performed and showed an increase in the tumor size to 21 × 19 × 15 cm. Four courses of VIP chemotherapy were given, and then a midline sternotomy with a resection of the large anterior mediastinal mass was done (Figure 2). Postsurgery, the patient was stable symptom-wise and a chest X-ray revealed no signs of pneumothorax.

**Figure 3:**
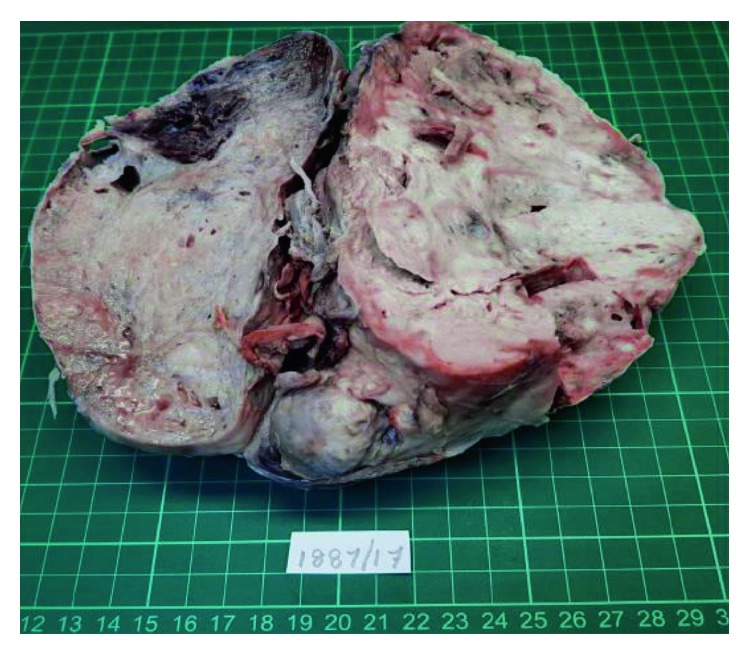
The cut surface of the mediastinal tumor is grey-white in appearance with areas of hemorrhage, necrosis, and cystic changes.

A 21 × 18 × 8 cm mediastinal mass weighing 2245 g was received in the lab for histopathological examination. The mass was encapsulated and nodular, with a greyish-white cut surface. Areas of necrosis, hemorrhage, and cystic spaces filled with mucoidal material were noted (Figure 3).

**Figure 4:**
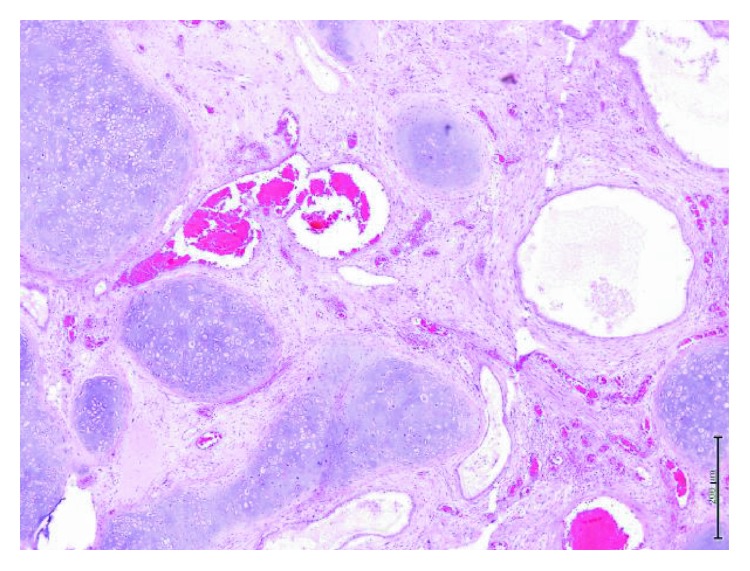
Low-power view of the tumor showing islands of mature cartilage, dilated blood vessels, and epithelial cysts within spindle stroma (H&E stain).

**Figure 5:**
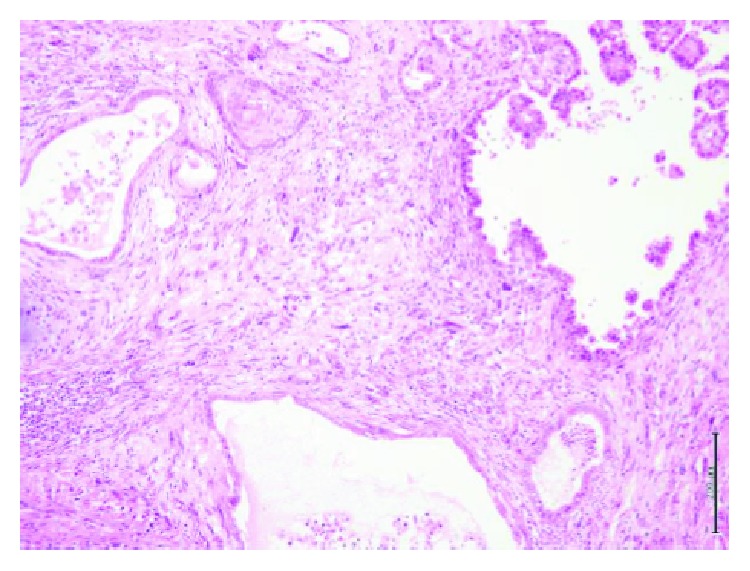
Image shows tumor composed of a mixture of epithelial cysts with papillary projections and squamous morules with sarcomatous stromal components (H&E stain).

**Figure 6:**
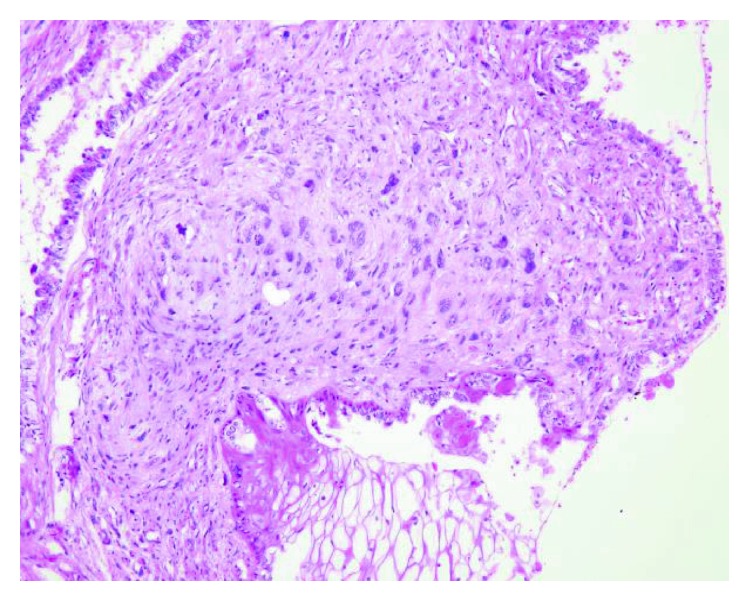
Image shows high-power microscopic appearance of the nonseminomatous germ cell tumor with sarcomatous changes (H&E stain).

Microscopic examination showed features of a malignant germ cell tumor consisting of differentiated and undifferentiated components. The differentiated component showed a mature teratoma composed of mature cartilage, bone trabeculae, smooth muscle fibers, and respiratory and gastric-type epithelia, while the undifferentiated component showed features of a yolk sac tumor containing hepatoid elements, proliferated sarcomatous spindle cells, and an increased mitotic rate (Figures 4–6).

Immunohistochemistry demonstrated a strong focal positivity for desmin in the stromal spindle and pleomorphic cells. The Ki-67 proliferating index was 20% in glandular and stromal cells. S100 was strongly positive in cartilaginous and focal stromal components. The tumor cells were negative for CD30, CD34, and SMA. The overall histomorphological and immunohistochemical appearances confirmed the diagnosis of a nonseminomatous germ cell tumor with sarcomatous changes (teratocarcinoma).

**Figure 7:**
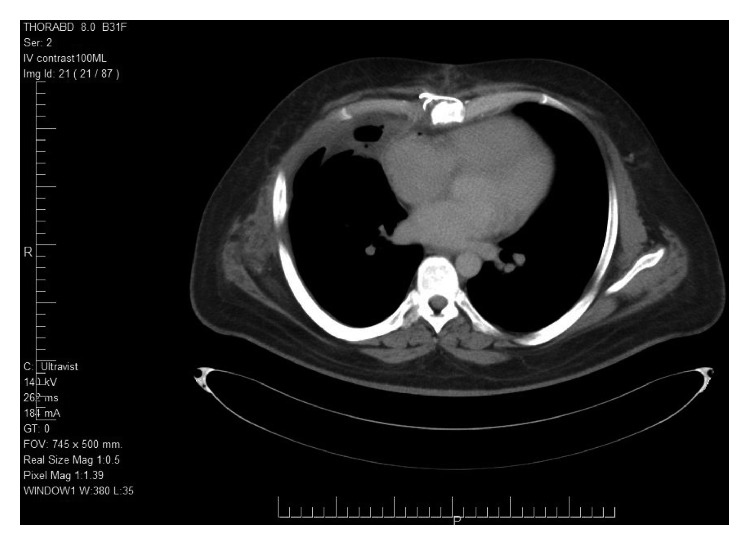
CT scan follow-up postresection of tumor.

Initially, blood investigations demonstrated elevated alpha-fetoprotein (AFP) at 18379.1 *μ*g/L (*N*: 0-9 *μ*g/L) and an elevated lactic dehydrogenase (LDH) at 399 U/L (*N*: 135-225 U/L). The patient's *β*-HCG level was normal. One month later, AFP increased to 19354.5 *μ*g/L and LDH increased to 460 U/L. Testosterone was also measured and was found to be slightly elevated at 49.93 nmol/L (*N*: 9.1-40). After the administration of chemotherapy, AFP levels were reduced to 32 *μ*g/L. After resection, the AFP level was at 0 *μ*g/L and the patient continued to regularly follow up with the oncologists, with regular measurement of the AFP level in every visit. Up until eighteen months following the resection of the tumor, AFP remained at 0 *μ*g/L. In addition, follow-up CT scan showed no residual tumor postresection (Figure 7).

## 3. Discussion

Patients with a large mediastinal mass are likely to present with symptoms of local compression including dyspnoea, dysphagia, and chest pain or tightness [3, 4, 6]. 85-90% of patients with NSGCTs are symptomatic as these tumors are usually invasive at the time of diagnosis [3, 5]. Eighty-five percent of NSGCTs are found in men with a mean age of presentation of 29 years. Almost 85-95% of patients have distant metastases at diagnosis; common locations include the lung, pleura, lymph nodes, and liver [3]. Our patient did not demonstrate any signs of metastasis either clinically or radiologically.

Some patients may demonstrate elevated serum levels of AFP or *β*-HCG, which may give a clue regarding an underlying germ cell tumor [3, 5]. Our patient demonstrated elevated levels of AFP initially, which normalized after tumor resection.

Generally, a chest CT scan permits accurate localization of a tumor and shows the extent of spread into adjacent structures. Additionally, it can identify the contents of a mass, which could include fat and calcification. Chest MRI can also be utilized to identify the constituents of the mass [1]. Our patient had a plain chest X-ray, CT scan, and MRI performed. The results of the MRI were mostly relied on to assess the dimensions and extent of invasion. Imaging of a NSGCT demonstrates a nonhomogeneous mass with areas of hemorrhage and necrosis [3, 5].

A teratocarcinoma is a malignant neoplasm derived from one or more of the three primary germ cell layers and contains components of embryonal carcinoma, seminoma, choriocarcinoma, or yolk sac tumor [3, 5]. Tumors such as adenocarcinoma, squamous cell carcinoma, and sarcoma can be associated with GCTs. Almost all mediastinal GCTs occur in the anterior mediastinum, and they constitute 10-15% of primary mediastinal tumors [2, 3]. Teratocarcinomas constitute 3-10% of mediastinal tumors and account for 1-5% of all germ cell neoplasms [7]. They usually appear as a lobulated mass with a thin capsule [3]. A teratocarcinoma describes the combination of a teratoma and embryonal carcinoma [4, 7]. The size of the mediastinal teratocarcinoma is varied, and Singhal and Jhavar report a giant primary mediastinal teratocarcinoma measuring 20.8 × 13 × 16 cm [4]. The tumor excised from our patient was slightly larger measuring 21 × 18 × 8 cm.

A mature teratoma has no gender preference. They are mostly cystic and encapsulated and adhere to adjacent structures by fibrous tissue. A yolk sac tumor is highly malignant. It is usually large and invasive when detected [2]. Since the tumor excised from our patient has a sarcomatous component, it can demonstrate a more aggressive behavior [2]. A tumor with a sarcomatous component can metastasize quickly and is known to be highly resistant to the standard combination chemotherapy for germ cell tumors [2].

The lungs are one of the most commonly invaded structures by mediastinal masses. In our patient, the right lung was compressed causing atelectasis of the anterior segment of the right upper lobe. The phrenic nerve and the superior vena cava can also be infiltrated by a tumor [1], but both structures remained intact and patent in our patient. It is an absolute contraindication to surgically resect a mediastinal tumor with direct invasion of the heart, trachea, or the great vessels [1]. The complete resection of a mediastinal GCT should be attempted because debulking proves to be of no benefit since residual GCT requires additional chemotherapy courses [3, 5].

Our case demonstrated a Ki-67 proliferation index of 80% prior to chemotherapy; however, this percentage dropped to 20% posttreatment. Yolk sac tumors are positive for AFP and negative for HCG [8], which was seen in our patient's mediastinal mass. A positive S100 was noted in the cartilaginous component in the specimen. Synaptophysin and chromogranin, as well as TTF1, were negative in our case, which excludes carcinoid and other lung malignancies. Calretinin was negative, which excludes mesothelioma. Tumors with a sarcomatous component are likely to stain positive for desmin, as in our case.

An appropriate chemotherapy regimen for a NSGCT is cisplatin-based chemotherapy. This can help improve patients' prognosis. Patients are treated every three weeks for a total of four courses [3]. Examples of chemotherapeutic agents that can be administered to patients with a NSGCT include cisplatin, vinblastin, etoposide, and bleomycin [2]. The standard of treatment is a combination of etoposide, bleomycin, and cisplatin (BEP) [3, 5]. Our patient received four courses of VIP chemotherapy. This combination includes etoposide, ifosfamide, and cisplatin (VIP). VIP is associated with myelotoxicity, which is one reason why most institutes prefer using BEP [9].

Mediastinal NSGCTs have a poor prognosis with only 40-50% of patients achieving complete remission [2, 3, 5]. Patients with NSGCTs have a 5-year survival of 48% which is much less than patients with a seminomatous GCT who have a survival rate of 86% [5]. Our patient demonstrated no signs of relapse within 18 months following the tumor resection. An elevated level of AFP can imply relapse [9]; thus, regular measurement during follow-up appointments in the clinic is recommended.

## 4. Conclusion

This report describes a giant NSGCT in a symptomatic male adult. NSGCTs can pose a challenge for cardiothoracic surgeons, oncologists, and pathologists. Prompt investigations including appropriate radiological imaging and histological studies are necessary for proper patient management and for improving the overall patient outcome.
